# Prevalence of risk factors for human immunodeficiency virus among sexually active women in Rwanda: a nationwide survey

**DOI:** 10.1186/s12889-023-17148-8

**Published:** 2023-11-10

**Authors:** Joseph Kawuki, Lilian Nuwabaine, Angella Namulema, John Baptist Asiimwe, Quraish Sserwanja, Ghislaine Gatasi, Elorm Donkor

**Affiliations:** 1 School of Nursing and Midwifery, Aga Khan University, Kampala, Uganda; 2 Jockey Club School of Public Health and Primary Care, Faculty of Medicine, The Chinese University of Hong Kong, Hong Kong, SAR, China; 3 Mbarara Regional Referral Hospital, Mbarara, Uganda; 4 Programmes Department, Relief International, Khartoum, Sudan; 5 Key Laboratory of Environmental Medicine Engineering, School of Public Health, Southeast University, Nanjing, 210009 Jiangsu Province China

**Keywords:** HIV risk factors, Sexually active women, Sexual risk behavior, Rwanda

## Abstract

**Background:**

The Human Immunodeficiency Virus (HIV) remains a global health burden, and despite the advancements in antiretroviral therapy and various strategies employed to curb HIV infections, the incidence of HIV remains disproportionately high among women. Therefore, this study aimed to determine the prevalence of the risk factors for the acquisition of HIV among sexually active women in Rwanda.

**Methods:**

Secondary data from the 2020 Rwanda Demographic Health Survey, comprising 10,684 sexually active women, was used. Multistage stratified sampling was employed to select the study participants. Multivariable logistic regression was conducted to determine the associated risk factors using the SPSS (version 25).

**Results:**

Of the 10,684 sexually active women, 28.7% (95% confidence interval (CI): 27.5–29.4) had at least one risk factor for HIV acquisition. Having no education (AOR = 3.65, 95%CI: 2.16–6.16), being unmarried (AOR = 4.50, 95%CI: 2.47–8.21), being from female-headed households (AOR = 1.75, 95%CI: 1.42–2.15), not having health insurance (AOR = 1.34, 95%CI: 1.09–1.65), no HIV test history (AOR = 1.44, 95%CI: 1.01–2.08), being from the poorest wealth quintile (AOR = 1.61, 95%CI: 1.14–2.27) and lack of exposure to mass media (AOR = 1.30, 95%CI: 1.07–1.58) were associated with higher odds of exposure to at least one HIV acquisition risk factor. In contrast, age groups of 25–34 (AOR = 0.56, 95%CI: 0.44–0.71) and 35–44 years (AOR = 0.62, 95%CI: 0.48–0.80), rural residence (AOR = 0.63, 95%CI: 0.49–0.81) and being from the western region (AOR = 0.67, 95%CI: 0.48–0.94) were associated with less odds of exposure to at least one HIV acquisition risk factor.

**Conclusion:**

More than a quarter of sexually active women in Rwanda had exposure to at least one risk factor for HIV acquisition. There is a need to maximize the use of mass media in disseminating HIV prevention and behavioral change messages. Engagement of religious leaders and promotion of HIV testing, especially among the never-testers, may be vital strategies in successful HIV prevention programs.

## Introduction

The Human Immunodeficiency Virus (HIV) remains a public health concern worldwide, causing negative health, social and economic consequences among women in developing countries [1, 2]. Globally, about 38 million people were living with HIV (PLWH) in 2022 [3], and approximately 50% of them were women [1, 4]. Africa shows a higher prevalence of HIV than any other continent, with an estimated average of 3.9% of its population living with HIV [5, 6]. In sub-Saharan Africa, out of a total of 33 million PLWH, 15.9 million were women of reproductive age, and approximately 5,000 women aged 15–24 years became infected with HIV every week [4–8].

Several studies have defined HIV acquisition risk factors as sexual activities that increase a person’s risk of acquiring sexually transmitted infections (HIV inclusive), such as unprotected sex and multiple sexual partners [9, 10]. In sub-Saharan Africa, more than 50% of rural women are exposed to early marriages, which limits their future opportunities for economic independence and decision-making and promotes gender-based violence, making them more vulnerable to multiple risks of exposure to HIV infection [11, 12].

Rwanda, which recorded an HIV prevalence of 3.0% in 2019 among people aged 15–49 years, had a higher proportion of HIV in women (3.6%) than in men (2.2%) [13]. Kigali, the capital city of Rwanda, had an HIV prevalence of 4.3% among the general population and 9.4% among women of reproductive age [14]. Although the HIV prevalence in Rwanda is still low compared to other East African countries like Uganda, at 5.7%, the HIV risk factors pose a great threat to the rise of new HIV infections, and if not addressed with caution, the HIV burden would be inevitable [13–15]. Currently, HIV is a disease with no cure but with medications to control and prevent its progression; hence, the best way to curb the epidemic is by preventing new infections, mainly through risk reduction.

Several studies conducted in Rwanda have indicated various factors that increase the risk of exposure to HIV infection, including sexual behaviours such as concurrent sexual partners, an earlier sex debut (< 19 years), and non-condom use [2]. In addition, women with a history of sexually transmitted infections (STIs) living in woman-headed households were more vulnerable to HIV infection [2]. Other studies in Rwanda showed that women who had experienced intimate partner violence (IPV) were 1.5 times more likely to be infected with HIV than women who had not experienced similar violence [13, 14]. Although the national HIV prevalence in Rwanda remains at 3%, province and place of residence are also key to HIV/AIDS risk in Rwanda [16]. The 2013–2014 Rwanda Biomedical Center report showed that key populations like female sex workers, their partners and clients, long-distance truck drivers, uniformed armed personnel, prisoners, and people who inject drugs play an important role in the dynamics of new HIV infections in Rwanda [17].

Apart from the well-known risk of Mother-to-Child Transmission (MTCT), HIV contributes to decreased fertility among PLWH by causing abortion and stillbirth, causes changes in their sexual and reproductive health behaviour, and also leads to life-threatening risks to both mother and unborn baby [14, 18]. Despite the substantial effect of HIV on child and maternal health, no study has explored the risk factors for HIV acquisition among sexually active women in Rwanda. Previous studies in the country have focused on HIV prevalence, testing, and associated factors [8, 14], or other aspects of HIV [13]. Other studies were focused on the risk factors for HIV acquisition either among the general population [7] or adolescents [19], but none among sexually active women.

This study, therefore, aimed to determine the prevalence of the risk factors for HIV acquisition and associated demographic factors among sexually active women in Rwanda using the 2020 Rwanda Demographic Health Survey. The study generates pivotal evidence that may inform policymakers and health professionals when formulating and implementing workable strategies to curb the spread of HIV.

## Methods

### Study sampling and participants

This analysis used the 2019–2020 Rwanda Demographic Health Survey (RDHS) data. The RDHS used a two-stage sample design; the first stage involved cluster selection of enumeration areas (EAs), and the second stage involved systematic sampling of 13,005 households in all the selected EAs [20]. The data used in this analysis were from household and women’s questionnaires.

The 2019–2020 RDHS data collection period was between November 2019 and July 2020 [20]. The women’s questionnaire collected data on women aged 15–49 years who were either permanent residents of the selected households or visitors who stayed in the household the night before the survey. Of the total 13,005 households that were selected for the survey, 12,951 were occupied, and 12,949 were successfully interviewed with a 99.9% response rate [20]. Additionally, from the selected households, 14,675 women aged 15–49 were eligible to be interviewed, but 14,634 women were successfully interviewed, giving a 99.7% response rate [20]. Given the study objectives and scope, this analysis, however, included only sexually active women in the last 12 months before the survey (n = 10,684). We excluded women who were not sexually active. We requested the RDHS dataset and obtained written permission for use from the MEASURE DHS website (URL: https://www.dhsprogram.com/data/available-datasets.cfm). Although the dataset contains hundreds of variables, only those relevant and applicable to our study were considered and used.

### Variables

#### Dependent variables

The outcome variable was risk factors for HIV acquisition, which was a composite variable constructed from four behaviours that expose women to HIV. These are: (i) Non-condom use during sex with the most recent partner among the unmarried (ii) Having multiple sexual partners in the last 12 months, (iii) Early sex debuts (below 18 years), and (iv) History of a sexually transmitted infection in last 12 months. The four items were first re-coded to yes or no, and the total scores for each individual were obtained. Individuals with at least one of the four risk factors were then coded yes, and otherwise no.

#### Independent variables

Based on the review of existing literature on risk factors for HIV [1, 21–25], we included possible determinants at individual, household, and community levels. Place of residence (categorized into rural and urban) and region (Kigali, South, West, East, and North) were the community-level factors included. For household-level factors, we considered household size (less than six, six and above), sex of household head (male, female), and wealth index (made of five quintiles ranging from the poorest to the richest quintile). Several individual-level factors were also included: age (15–24, 25–34, 35–44, 45–49), educational level (no education, primary, secondary, tertiary), working status (yes, no), marital status (married, unmarried), religion (Catholic, protestant, Adventist, “Others”). “Other” religions included Moslem, Jehovah’s Witness, health insurance (yes, no), exposure to mass media (yes, no), mobile phone ownership (yes, no), internet usage (yes, no), comprehensive knowledge of HIV (yes, no), and HIV test history (yes, no). The wealth index was calculated by RDHS from information on household asset ownership using principal component analysis and categorized into five quintiles that ranged from the poorest to the richest quintile [26]. Media exposure was measured as a woman having access to any of these: radio, newspapers, and television. Comprehensive knowledge of HIV was assessed using six items: three on HIV prevention information and three questions on misconceptions of HIV transmission modes.

### Statistical analysis

Frequency distributions were used to describe the background characteristics of the respondents, where we presented frequencies and proportions/ percentages for categorical dependent and independent variables. We, then, conducted bivariable logistic regression to assess the independent association of each predictor variable with the outcome variable, and we presented the respective crude odds ratio (COR), 95% confidence interval (CI) and p-values. Multivariable logistic regression was then conducted, including independent variables found to be significant at a p-value < 0.25 in bivariable analysis. Variables reported to be significantly associated with risk factors for HIV in previous studies were also included in the multivariable model, regardless of their significance in bivariable analysis. Respective adjusted odds ratios (AOR), 95%CI and p-values were obtained and presented, with a statistical significance of < 0.05. To account for the unequal probability sampling in different strata and ensure the representativeness of the study results, DHS sample weights were applied [27, 28]. We used Statistical Package for Social Sciences (SPSS) (version 25.0)- complex samples package. Individual sample weight, sample strata for sampling errors/design, and cluster number were incorporated into the analysis plan to account for the multistage sample design intrinsic to the RDHS dataset [27, 28]. Multi-collinearity was also assessed among all the predictor variables in the model using a variance inflation factor (VIF) of less than 10 as a cutoff [29]. None of the factors exceeded the cutoff.

## Results

### Characteristics of participants

The analysis comprised 10,684 sexually active women (Table 1). The majority were aged 25–44 years (69.2%), working (75.4%), married (69.3%), had primary education (63.3%), had health insurance (82.1%), and resided in rural areas (80.2%). Moreover, the majority were from male-headed households (70.5%) of less than 6 members (64.0%), had exposure to mass media (80.8%), did not use the internet (87.6%), had comprehensive knowledge about HIV (66.1%) and had ever tested for HIV (93.5%), Table 1.

**Table 1:** Background characteristics of sexually active women as per the 2020 Rwanda Demographic Health Survey

Characteristics	Frequency (%), N = 10,684
**Age in years**	
15–24	2090(19.6)
25–34	3895(36.5)
35–44	3498(32.7)
45–49	1200(11.2)
**Education level**	
Tertiary	490(4.6)
Secondary	2116(19.8)
Primary	6764(63.3)
No education	1314(12.3)
**Working status**	
Working	8059(75.4)
Not working	2625(24.6)
**Marital status**	
Married	7401(69.3)
Unmarried	3282(30.7)
**Religion**	
Catholic	3894(36.5)
Protestant	5029(47.1)
Adventist	1353(12.7)
Others	408(3.8)
**Health insurance**	
Yes	8768(82.1)
No	1916(17.9)
**Wealth index**	
Richest	2305(21.6)
Richer	2133(20.0)
Middle	2004(18.8)
Poorer	2030(19.0)
Poorest	2212(20.7)
**Residence**	
Urban	2110(19.8)
Rural	8574(80.2)
**Region**	
Kigali	1610(15.1)
West	2268(21.2)
East	2929(27.4)
North	1622(15.2)
South	2254(21.1)
**Sex of household head**	
Male	7532(70.5)
Female	3152(29.5)
**Household size**	
Below 6	6843(64.0)
Above 6	3841(36.0)
**Exposure to mass media**	
Yes	8629(80.8)
No	2055(19.2)
**Mobile phone ownership**	
Yes	5455(51.1)
No	5229(48.9)
**Internet use**	
Yes	1328(12.4)
No	9356(87.6)
Comprehensive HIV knowledge*	
Yes	7060(66.1)
No	3615(33.8)
**HIV test history**	
Yes	9989(93.5)
No	695(6.5)

STI = sexually transmitted infection, HIV = human immunodeficiency virus, * =9 missing values

**Risk factors for HIV among sexually active women as per the 2020 Rwanda Demographic Health Survey**.

Regarding risk factors for HIV, 28.7% (95%CI: 27.5–29.4) had at least one risk factor, of which early sex debuts (25.4%) and having multiple sex partners (12.1%) were the most common. This was followed by non-condom use among the unmarried (9.8%) and STI history (4.4%), as detailed in Table 2.

**Table 2:** Risky factors for HIV among sexually active women as per the 2020 Rwanda Demographic Health Survey

Risk factor	Frequency, N = 10,684	Prevalence, %	95% Confidence interval
Non-condom uses at last intercourse among unmarried ^a^	843	9.8*	9.2–10.4
Multiple sex partners	1297	12.1	11.6–12.8
STI history in the last 12 months	475	4.4	4.1–4.9
Early sex debut (below 18 years)	2713	25.4	24.5–26.0
At least one of the above risk factors	3071	28.7	27.5–29.4

STI = sexually transmitted infection, HIV = human immunodeficiency virus

a = missing 2071 responses, hence, the denominator was 8613

*= prevalence is 7.9%, while using 10,684 as the denominator

### Factors associated with risk factors for HIV among sexually active women in Rwanda

The results of the bivariable analysis are detailed in Table 3, with significant factors having an independent association with risk factors for HIV acquisition highlighted. After multiple logistic regression, the factors with statistically significant associations included age, educational level, wealth index, marital status, religion, health insurance, wealth index, residence, sex of household head, exposure to mass media, and HIV test history (Table 3).

Compared to women having tertiary education, those with secondary (AOR = 1.94, 95%CI: 1.21–3.09), primary (AOR = 2.47, 95%CI: 1.55–3.95) and no education (AOR = 3.65, 95%CI: 2.16–6.16) had higher odds of exposure to at least one HIV risk factor. The unmarried women (AOR = 4.50, 95%CI: 2.47–8.21) also had higher odds of exposure to at least one risk factor for HIV acquisition compared to the married, similar to those from female-headed households (AOR = 1.75, 95%CI: 1.42–2.15) who also had higher odds than those from male-headed households. Compared to the Catholics, the Protestants (AOR = 1.28, 95%CI: 1.09–1.50) and other religions (AOR = 1.99, 95%CI: 1.41–2.80) had higher odds of exposure to at least one risk factor for HIV acquisition, same as those with no health insurance (AOR = 1.34, 95%CI: 1.09–1.65) who also had higher odds compared to those having health insurance. Women in the poorer (AOR = 1.57, 95%CI: 1.13–2.17) and poorest (AOR = 1.61, 95%CI: 1.14–2.27) wealth index had higher odds of exposure to at least one HIV risk factor compared to those in the richest wealth index. Similarly, those with no exposure to mass media (AOR = 1.30, 95%CI: 1.07–1.58) and no HIV test history (AOR = 1.44, 95%CI: 1.01–2.08) had higher odds of exposure to at least one HIV acquisition risk factor compared to those with media exposure and HIV test history, respectively.

In contrast, compared to women aged 15–24 years, those of 25–34 years (AOR = 0.56, 95%CI: 0.44–0.71) and 35–44 years (AOR = 0.62, 95%CI: 0.48–0.80) were 44% and 38% less likely to have HIV risk factors, respectively. Furthermore, rural residents (AOR = 0.63, 95%CI: 0.49–0.81) and those from the western region (AOR = 0.67, 95%CI: 0.48–0.94) had 37% and 33% less odds of exposure to at least one risk factors for HIV, compared to their respective urban and Kigali counterparts, (Table 3).

**Table 3:** Factors associated with risk factors for HIV among Rwandan women as per 2020 RDHS

Characteristics	Crude odds ratio (95%CI)*	P-value	Adjusted odds ratio (95%CI)**	P-value
**Age**		**< 0.001**		**< 0.001**
15–24	1		1	
25–34	0.28(0.24–0.33)		**0.56(0.44–0.71)**	
35–44	0.24(0.21–0.28)		**0.62(0.48–0.80)**	
45–49	0.26(0.21–0.32)		0.74(0.55–1.01)	
**Education level**		**< 0.001**		**< 0.001**
Tertiary	1		1	
Secondary	2.31(1.66–3.21)		**1.94(1.21–3.09)**	
Primary	1.65(1.20–2.28)		**2.47(1.55–3.95)**	
No education	1.85(1.31–2.62)		**3.65(2.16–6.16)**	
**Working status**		**0.025**		0.919
Working	1		1	
Not working	1.17(1.02–1.34)		0.99(0.82–1.19)	
**Marital status**		**< 0.001**		**< 0.001**
Married	1		1	
Unmarried	6.06(3.39–10.83)		**4.50(2.47–8.21)**	
**Religion**		**< 0.001**		**< 0.001**
Catholic	1		1	
Protestant	1.02(0.91–1.15)		**1.28(1.09–1.50)**	
Adventist	0.92(0.78–1.09)		1.16(0.91–1.47)	
Others	1.78(1.37–2.31)		**1.99(1.41–2.80)**	
**Health insurance**		**< 0.001**		**0.005**
Yes	1		1	
No	1.62(1.41–1.87)		**1.34(1.09–1.65)**	
**Wealth index**		**0.001**		**0.045**
Richest	1		1	
Richer	0.98(0.82–1.18)		1.23(0.90–1.68)	
Middle	0.83(0.67–1.02)		1.31(0.96–1.79)	
Poorer	1.06(0.87–1.30)		**1.57(1.13–2.17)**	
Poorest	1.25(1.03–1.50)		**1.61(1.14–2.27)**	
**Residence**		**< 0.001**		**< 0.001**
Urban	1		1	
Rural	0.63(0.53–0.75)		**0.63(0.49–0.81)**	
**Region**		**< 0.001**		**< 0.001**
Kigali	1		1	
West	0.58(0.46–0.74)		**0.67(0.48–0.94)**	
East	0.86(0.68–1.08)		1.18(0.85–1.64)	
North	0.54(0.41–0.69)		0.70(0.49–1.02)	
South	0.68(0.54–0.86)		0.84(0.60–1.18)	
**Sex of household head**		**< 0.001**		**< 0.001**
Male	1		1	
Female	6.99(6.15–7.96)		**1.75(1.42–2.15)**	
**Household size**		0.123		0.084
Below 6	1		1	
Above 6	1.10(0.98–1.23)		1.17(0.98–1.39)	
**Exposure to mass media**		**0.003**		**0.008**
Yes	1		1	
No	1.23(1.07–1.41)		**1.30(1.07–1.58)**	
**Mobile phone ownership**		**< 0.001**		0.056
Yes	1		1	
No	0.75(0.67–0.85)		0.83(0.68–1.01)	
**Internet use**		**0.022**		0.809
Yes	1		1	
No	0.78(0.64–0.97)		0.96(0.66–1.39)	
**Comprehensive HIV knowledge**		0.082		0.863
Yes	1		1	
No	1.10(0.99–1.23)		0.99(0.85–1.15)	
**HIV test history**		**< 0.001**		**0.047**
Yes	1		1	
No	2.45(1.96–3.07)		**1.44(1.01–2.08)**	

**Bold** = significant, *= significant at 0.25, ** = significant at 0.05, CI = confidence interval, RDHS = Rwanda demographic health survey, STI = sexually transmitted infection, HIV = human immunodeficiency virus

## Discussion

A secondary analysis of RDHS data to determine the prevalence of HIV risk factors and associated socio-demographics among sexually active women was conducted. The prevalence of at least one of the four risk factors for HIV acquisition was found to be 28.7% among sexually active women. This is similar to the HIV risk factor prevalence reported in Brazil (28.7%) [30] but lower than that in Sierra Leone (38.1%) [1] and Togo (34.6%) [31]. The observed variations in the prevalence of the risk factors for HIV acquisition may be attributed to the differences in efforts made to sensitize the target population about HIV prevention, as well as the socio-demographics across countries [1]. A 28.7% prevalence of HIV risk factors is large, which may depict a gap in HIV/AIDS prevention and control strategies. Therefore, there is a great need to strengthen health promotion and behavior change programs among sexually active women.

In this study, the four risk factors for HIV acquisition included non-condom use at last intercourse among unmarried women, having multiple sex partners, STI history in the last 12 months, and early sex debuts. The study results showed that 25.4% of the women had early sex debuts, 12.1% had multiple sexual partners, 9.8% did not use condoms yet were unmarried, and 4.4% had an STI history in the last 12 months. These study findings are supported by other studies conducted in Zambia [21] and Sierra Leone [1], which reported women having multiple sexual partners as a risk factor for HIV acquisition. On the other hand, these studies conducted in Zambia [21] and Sierra Leone [1] found that 37.8% and 27.4% of sexually active women, respectively, had not used condoms at their last sexual intercourse, which was higher than the 9.8% non-condom use prevalence in Rwanda. The reported 12.1% prevalence of multiple sexual partners in this study is higher than that in Sierra Leone (4.8%) [1], but lower than the one reported in Zambia (1.9%) [21]. Moreover, the reported 24.5% prevalence of early sex debut in this study is higher than the 10.1% in Tanzania and 10.4% in Uganda [32]. The observed differences in the prevalence of the risk factors for HIV acquisition between this study and other countries in Africa may be due to gaps in HIV/AIDS prevention and control strategies implemented in different countries [33]. The differences in cultural norms, health policies, and legislation across countries may also explain the differences.

This study also assessed several socio-demographics associated with the prevalence of HIV risk factors, where age, residence, religion, sex of household head, marital status, working status, education level, wealth index, health insurance, and history of HIV testing were significant correlates. Sexually active women aged 25–34 years and 35–44 years had lower odds of risk factors for HIV infections compared to the young age group of 15–24 years. The findings are similar to those of other studies in Swaziland [34], Sierra Leone [1], Zambia [21], and China [35]. This may be because older women 25–34 and 34–44 years old tend to be more informed about sexual matters and more knowledgeable about HIV [19], whereas younger women are generally explorative and easily fall victim to peer pressure, hence easily indulging in sexual behaviors associated with an increased risk of HIV acquisition [1, 35].

In this study, it was observed that women with no education were more likely to indulge in sexual behaviours associated with a high risk of HIV infection compared to those with some level of education, and the risk factors for HIV acquisition were lowest in women who had attained tertiary education. Women with no formal education are most likely to have limited knowledge about HIV [19]. They also tend to lack formal employment and may be completely dependent on male sexual partners for financial support, making them unable to negotiate safer sex practices [21, 35]. The study finding is consistent with a study in Brazil that reported that the higher the education level, the lower the indulgence in sexual behaviours associated with an increased risk of HIV acquisition [35–37].

This study showed that women-headed households and unmarried women are associated with higher odds of HIV risk factors compared to their respective counterparts. The findings are consistent with a study from Sierra Leone [1] that also reported an association between sexual behaviors linked with an increased risk of HIV acquisition, with being unmarried and living in households headed by women. Unmarried women from female-headed households generally tend to have freedom from marital obligations and are more likely to participate in sexual networks for their survival and economic reasons [34, 38].

It was observed that sexually active women belonging to Protestant and “Other” religions (Moslem, Jehovah’s Witness) had higher odds of indulging in sexual behaviours associated with a high risk of HIV infection compared to Catholics. The findings are consistent with a study in Rwanda [39] which reported that Catholic respondents were less likely to participate in sexual risk behaviour because they had more comprehensive HIV knowledge due to HIV awareness messages and HIV testing encouragement compared to their protestant counterparts. The finding may be attributed to the different beliefs, values, and norms associated with various religions [40, 41]. Therefore, continuous engagement of religious and cultural leaders in HIV prevention programs or campaigns is highly recommended.

Sexually active women in poorer and poorest wealth quintiles had higher odds of HIV risk factors compared to those in the richest quintile, similar to sexually active women with no health insurance, who also had higher odds compared to those with health insurance. The study findings are consistent with a study in Rwanda [19], which reported that poor women had limited access to media and hence little knowledge about HIV. Economically disadvantaged women may indulge in sex work or depend on their male sexual partners for economic survival as they may not have formal employment. Additionally, due to poverty, they may not afford health insurance nor access to good healthcare services [1, 42].

Place of residence was also associated with HIV risk factors, where women residing in urban areas had higher odds of risk factors for HIV infection compared to those in rural areas. The study findings are consistent with studies in Sierra Leone [21] and China [35] which reported that women in urban areas had a greater number of HIV infection risk factors. This may be because women in urban areas are exposed to a high population density, with risky populations like long-distance truck drivers included, hence a high likelihood of indulging in sexual behaviours associated with an increased risk of HIV acquisition [36, 43]. Moreover, region was also associated with risk factors for HIV acquisition, where the western region, compared to Kigali, had lower odds of HIV risk factors compared to other regions. The western province is a rural area with less activity and entertainment places like bars, where women are less likely to participate in sexual behaviours associated with an increased risk of HIV acquisition [19]. The study findings are consistent with studies in Eswatini [44], which reported that women in rural areas are likely to have a limited number of lifetime sexual partners, hence reducing the risk for HIV.

Sexually active women who were not exposed to mass media had higher odds of indulging in sexual behaviours associated with a high risk of HIV infection acquisition compared to those exposed to mass media. The study findings are consistent with a study in Ghana that reported a reduction in HIV acquisition risk factors among people who had a TV or radio in their homes [45]. This may be because the HIV/AIDS organizations and the Ministry of Health, Rwanda disseminate HIV information via newspapers, radio, and TV [19]. Therefore, media should be explored further with more innovative strategies to continue providing adequate knowledge about HIV prevention and control, which enables the adoption of appropriate attitudes and practices by an even wider audience [19, 45].

In this study, the history of HIV testing was associated with risk factors for HIV, whereby women with a history of HIV testing had lower odds of indulging in sexual behaviours associated with an increased risk of HIV acquisition. The study findings are consistent with a study in Rwanda that reported that HIV testing was associated with a decrease in indulging in HIV risk factors [13]. This may probably be due to the pretest and post-test counselling and education on safer sex practices provided during HIV testing that non-testers might miss if no alternative avenues are offered [46].

### Strengths and limitations

This study is a secondary data analysis of a large weighted sample of 10,684 sexually active Rwandan women from the current DHS, therefore, our findings are generalizable to all sexually active women in Rwanda. However, our study is not without limitations. There is also a possibility of recall bias due to self-reported answers and false answers due to social desirability and the sensitive nature of the study topic. Missing data was inevitable on some key risk factors, such as transactional sex, so we could not include it in the analysis. The cross-sectional nature of the study limits the capacity to attribute causality to the findings. Moreover, there was a lack of data on other key sexual risk behaviours such as inter-generational sex partnerships and vital correlates of HIV risk factors such as alcohol and drug use during or before sex. In addition, due to limited data availability in the DHS dataset, we could not explore the impact of broader structural and social drivers of the HIV epidemic, such as criminalization of key population groups and pre-exposure prophylaxis availability, among others. These should be explored in future research since behaviour change interventions alone have a limited impact on the control of the HIV epidemic. Despite the limitations, the study provides valuable information on HIV risk factors among sexually active women in Rwanda.

## Conclusions

The study showed that over a quarter of the sexually active women in Rwanda had encountered at least one sexual behaviour associated with an increased risk of HIV acquisition. Age, residence, region, sex of household head, marital status, HIV testing history, wealth index, health insurance, and exposure to mass media were associated with HIV acquisition risk factors in Rwanda. There is a need to maximize the use of mass media in disseminating HIV prevention and behavioral change messages, taking into account the individual and household dynamics/ variations. Furthermore, the engagement of religious leaders in HIV prevention programs would be vital, as would the promotion of HIV testing, especially among the never-testers, as it is another avenue for HIV prevention, education, and counselling.

## Data Availability

The data set used in this study is openly available upon permission from the MEASURE DHS website (URL: https://www.dhsprogram.com/data/available-datasets.cfm). However, authors are not authorized to share this data set with the public but anyone interested in the data set can seek it with written permission from the MEASURE DHS website (URL: https://www.dhsprogram.com/data/available-datasets.cfm).

## Abbreviations

AIDS: Acquired Immunodeficiency Syndrome
AOR: Adjusted Odds Ratio
ART: Antiretroviral therapy
CI: Confidence Interval
COR: Crude Odds Ratio
DHS: Demographic Health Survey
EA: Enumeration area
HIV: Human Immunodeficiency Syndrome
MTCT: Mother-to-Child Transmission
OR: Odds Ratio
PLWH: People Living with HIV
RDHS: Rwanda Demographic Health Survey
SPSS: Statistical Package for Social Science
SSA: Sub Saharan Africa
VIF: Variance Inflation Factor
